# A Genomic and Phenotypic Investigation of Feed Efficiency and Growth Traits in Targhee and Rambouillet Sheep

**DOI:** 10.3390/ani15060783

**Published:** 2025-03-10

**Authors:** Daniel Schaub, Christian J. Posbergh

**Affiliations:** Department of Animal and Range Sciences, Montana State University, Bozeman, MT 59717, USA; daniel.schaub2@student.montana.edu

**Keywords:** *DMRT2*, feed efficiency, genetics, growth, maintenance, RFI

## Abstract

The U.S. range sheep industry uses estimated breeding values (EBVs) as part of their breeding objectives to increase post-weaning weight. The study objective was to quantify the relationship between lamb growth EBVs, feed intake, and feed efficiency. Eighty-one range ewe lambs were enrolled in the study to measure residual feed intake (RFI) over two 42-d periods at both the weaning and yearling stages. The ewe lambs’ post-weaning weight EBVs (PWWT EBVs) were linearly associated with their phenotypic traits. Preliminary genome wide associations (GWAs) were also performed with Dry Matter Intake (DMI), RFI, mid-test body size, and average daily gain (ADG) and Ovine 50K SNP genotypes. Post-weaning weight EBVs were associated with dry matter intake (DMI) (*p* < 0.05) but had no association with residual feed intake (RFI) (*p* > 0.05) in both experimental periods. However, PWWT EBV was predictive of mid-test body weight in both periods (*p* < 0.05). A single SNP at Oar2:68,812,505, located within *DMRT2*, was associated with DMI and RFI in the second experimental period (Bonferroni corrected *p* <0.05). While selecting for higher post-weaning weight range ewes may increase feed consumed due to a larger body size, it was not associated with feed efficiency.

## Simple Summary

Over the past forty years, United States sheep producers have selectively bred range sheep for larger post-weaning weights using estimated breeding values as part of their breeding objectives. However, producers have observed increases in feed intake, prompting concern that higher weaning weights were inadvertently lowering feed efficiency of their animals. To test this hypothesis, 81 ewe lambs were enrolled in a feed efficiency trial to measure residual feed intake during growth and maintenance phases and genotyped to determine the relationships between these traits. A single genetic marker within *DMRT2* was associated with feed efficiency, warranting further investigation into its possible role in feed efficiency and growth. No relationship was observed between feed efficiency and weight estimated breeding values, indicating range sheep producers have not indirectly reduced feed efficiency as they selected for higher weights.

## 1. Introduction

Feed costs are one of the largest expenses sheep producers pay comprising upwards of 60% of total costs [1]. Therefore, improving feed efficiency is a top priority of many animal industries to reduce feed consumption and its associated costs while retaining production. However, despite the economic importance, minimal research has been published investigating sheep feed efficiency genetics and there is no estimated breeding value (EBV) for feed efficiency in U.S. sheep populations.

Western U.S. sheep producers select for larger weaning weights rather than feed efficiency to generate faster growing lambs. For example, from 1978 to 2005, weaning weight increased from 28.6 kg to 37.2 kg in the Rambouillet sheep breed [2]. Faster growing lambs are important to producers since they typically reach market weight at a younger age, thereby reducing feed costs associated with keeping lambs longer to reach harvest size. To achieve this faster growth, some producers use the National Sheep Improvement Program to estimate breeding values for weight traits that enable more accurate selection for higher weaning weights [3]. The use of (PWWT) post-weaning weight EBVs is a common tool used in the Rambouillet and Targhee breeds to select for heavier lambs.

Residual feed intake (RFI) is commonly used in other livestock species, such as beef cattle and swine, to measure and improve feed efficiency [4,5]. Residual feed intake effectively identifies animals that consume less feed without affecting performance [6]. Several studies have described RFI across different sheep breeds and management systems but few have investigated links to genetic markers or estimated breeding values [7,8,9,10,11,12,13,14,15].

Previous genetic approaches investigating sheep feed efficiency include heritability estimates and genome wide association studies (GWAS). Heritability estimates show feed efficiency to be low to moderately heritable in sheep with narrow sense heritability estimates ranging between 0.11 and 0.46 [16,17,18]. Due to the low to moderate heritability estimates, the use of genetic and genomic tools is likely needed to make genetic progress toward improved feed efficiency compared to direct selection. Genome wide association studies are an important tool to find associations between single nucleotide polymorphisms (SNP) and phenotypes of interest. Cockrum et al. (2012), performed a GWAS and found that ovine chromosomes 1, 2, 3, and 18 had the greatest number of SNPs associated with RFI and identified *GLIS1*, *IL1RAPL1*, *SOX5*, and *SOX6* as candidate genes [19]. Pasandideh et al. (2018), performed a GWAS and identified MAGI1 and ARRDC3 was associated with lower feed efficiency in 6–9 month old lambs and that PPP4R2 and FOXP1 were associated with lower feed efficiency in 9–12 month old lambs [20].

The relationship between PWWT EBVs and feed efficiency in sheep has not been reported. If the relationship between the two measurements is negative, sheep producers could be harming their profitability by indirectly selecting less feed efficient sheep. Therefore, quantifying the relationship between lamb growth EBVs, feed intake, and feed efficiency is crucial for improving the sheep industry’s profitability. Therefore, the objectives of the study were to: (1) characterize feed intake, average daily gain, and feed efficiency over growth and maintenance production phases, (2) quantify the relationship between PWWT EBVs, feed intake, and feed efficiency, and (3) to identify genome wide associations with feed efficiency.

## 2. Materials and Methods

All procedures and protocols utilizing these animals were approved by Montana State University Animal Care and Use Committee (Protocol no. 2020-AA13).

### 2.1. Study Population

Purebred and NSIP enrolled Targhee (n = 40) and Rambouillet (n = 41) ewe lambs born in 2021 were enrolled from the Montana Agricultural Experiment Station flock. Lambs were selected to exhibit variation in each breed’s PWWT EBVs in our study population.

Two experimental periods were conducted to determine daily feed intake and average daily weight gain of the ewe lambs at different physiological stages. The Vytelle system was used for feeding and it weighs feed in the bunk, records the RFID tags of the lambs, and measures feed disappearance. Elevated platforms and elevated bunks were used to allow sheep access to the feed bunks since they were designed for cattle height.

### 2.2. Experimental Period 1

All procedures were performed at Montana State University’s Fort Ellis Research Farm (45°39′57″ N, 110°58′24.3″ W) located in Bozeman, Montana. On 30 August 2021, a 56-day feeding experimental period began consisting of a 14-day adaptation period and a 42-day data collection period. Forty-two days is a standard benchmark for measuring feed efficiency in sheep [17]. Ewe lambs (121 ± 5 days old) were equally separated into four pens, stratified by breed and initial body weight. Each pen contained two feed bunks. Ewe lambs were given ad libitum access to an alfalfa-based pellet and water (Table 1). During the adaptation period, seven ewe lambs were removed from the study due to poor adaptation of the Vytelle system. Lambs were considered poorly adapted if they lost body weight or failed to eat from the feed bunks for more than two consecutive days. Ewe lambs were weighed at the beginning and end of the experimental period, as well as weekly throughout the experimental period. Feed samples were taken and sent to a commercial laboratory (Dairy One, Ithaca, NY, USA) for nutrient analysis.

### 2.3. Experimental Period 2

On 15 April 2022, a second feeding experimental period began with 70 ewes from the same set of animals from experimental period one, consisting of a 10-day adaptation period and a 42-day data collection period. A 10-day adaptation period was determined to be enough time as the ewes have already experienced the Vytelle system. Yearling ewes (355 ± 5 days old) were equally separated into two pens stratified by breed and initial body weight. Each pen contained four feed bunks. Ewes were given ad libitum access to an alfalfa hay-based maintenance diet and water (Table 1). Yearling ewes were weighed at the beginning, end, and weekly to collect throughout the experimental period. Feed samples were taken and sent to a commercial laboratory (Dairy One, Ithaca, NY, USA) for nutrient analysis.

### 2.4. Diet Compositions

The chemical compositions of the alfalfa pellet and hay diets are listed in Table 1.

**Table 1:** Chemical Composition of Diets used in Experimental Period One and Two on a Dry Matter Basis.

Components (%)	Alfalfa Pellets	Alfalfa Hay
Dry Matter	90.6	89.7
Crude Protein	17.7	17.4
ADF	32.7	31.1
aNDF	41.7	37.2
TDN	61	63

### 2.5. Feed Intake and Trait Analysis

Daily feed intakes for ewes were exported by using the Process Intakes and Export Behavior Data routine of the Vytelle Data Acquisition software. The ADG of individual ewes was calculated through a calculation of the slope of a linear regression of weight recorded over the experimental period. Regression coefficients of this linear regression were then used to calculate mid-test body weight (MTBW). Mid-test metabolic body weight (MBW) was then calculated using the following equation:

MTBW^0.75(1)

Raw feed intake data was transformed into a dry matter intake (DMI) basis. Daily DMI estimates were then modeled via linear regression of each ewe’s dry matter feed intake over the days of the experimental period. All feed intake data analysis was performed using R 4.2.1 and R-Studio Build 461 (R Core Team, 2022; RStudio Team, 2023).

Expected feed intake was modeled for each ewe through the following equation:

Expected Feed Intake = DMI regression intercept + B1 ∗ MBW + B2 ∗ ADG + error.(2)

Linear regressions on DMI over ADG and MTBW were then calculated to determine coefficients B1 and B2, respectively. Ewe RFI was then calculated for each ewe by taking the difference between their actual feed intake and expected feed intake. Phenotypic correlation coefficients were calculated between animal weights, PWWT, feed intake and efficiency data for experimental period 1, 2, and between periods 1 and 2. Repeatability for RFI was calculated using the rptR package in R [21]. Significance for correlations was set at *p* < 0.05.

### 2.6. Genomic Analysis

Tissue sampling units were taken from the ear of selected ewe lambs at birth and stored for subsequent DNA extraction and genotyping. Sixty-seven ewes were genotyped on the GGP Ovine 50k SNP chip (Neogen Geneseek, Lincoln, NE, USA). Genotypes were uploaded into Golden Helix SNP and Variation Suite (v8.9.1, Bozeman, MT, USA) for processing and analysis. Genome coordinates are from the Oar v3.1 assembly. Quality controls were applied and SNPs were removed if they had a call rate of less than 90%, more than two alleles, or a minor allele frequency of less than 0.05. Individuals were removed if they had a call rate less than 0.90. These thresholds removed 7033 SNPs leaving 44,855 SNPs and 67 individuals available for subsequent analysis. Genome wide associations were conducted using EMMAX to fit the genomic relationship matrix as a random effect to adjust for potential population structure in the dataset [22]. Dry matter intake, ADG, RFI, and MTBW phenotypes from both experimental periods were used as the variables of interest for the GWAS. A Bonferroni corrected *p*-value of 0.05 was set as the threshold for genome-wide significance. Genes were considered candidate genes if they were within 1 Mb window surrounding any associated marker.

## 3. Results and Discussion

The median age for the ewes was 121 ± 5 and 355 ± 5 days at the start of each experimental period for periods one and two, respectively. Between the two experimental periods MTBW, MBW, and ADG differed as expected (*p* < 0.05, Table 2). Sheep in experimental period two had an average MTBW 15.1 kg higher than experimental period one. Ewes in experimental period one gained 0.26 ± 0.12 kg per day compared to experimental period two, which gained −0.02 ± 0.16 kg per day, indicative of a maintenance physiological state. Other production measurements by experimental period are listed in Table 2.

### 3.1. Trait Correlations

Phenotypic correlations between traits in experimental period 1 and period 2 are found in Table 3. In experimental period 1, as expected, bodyweight correlated with DMI (0.783) and with PWWT EBV (0.438). Positive correlations between DMI and PWWT EBV (0.280) and RFI (0.619) were also detected. These correlations are similar to what has been previously reported for growing lambs [6,23,24]. The lack of correlation between PWWT EBV and RFI indicates that producers are not selecting on feed efficiency when they are selecting lambs with a higher PWWT EBV.

However, no correlation was detected between PWWT EBV and ADG. This may be a result of feeding ewes and measuring ADG at a later stage of the growth curve, and as such there may not be as strong of a relationship between ADG during approximately 120 through 176 days of age and PWWT EBV which is meant to represent weight at approximately 120 days of age. While weaning weight is correlated with mature size and body weight is typically associated with ADG, when producers use PWWT EBVs to make selection decisions, they should expect to see larger faster, growing lambs that reach higher weights at weaning [25].

As observed in this study ewes with larger body weights had larger DMI, something that carries over to maturity and may lead to higher ewe flock maintenance costs and limit flock profitability. Increasing DMI in sheep for producers who live in forage-limited areas resulted in a reduction of flock size to meet nutrient requirements, which limits revenue by reducing future lamb crop sizes [26]. In simulations, most negative economic effects from increasing weaning weight were a consequence of larger ewes, due to the positive correlation between yearling weight and weaning weight, but this impact of yearling weight was varied depending on resource availability and forage price [26]. Selection towards higher weaning weights without regard to feed efficiency may be more advantageous when forage cost is low, feed resources are not limited, and markets do not discount over-finished lambs.

In experimental period 2, bodyweight correlated with ADG (0.387), DMI (0.562), and PWWT EBV (0.330). A correlation between RFI and DMI (0.820) was also detected but no correlations were detected between RFI and bodyweight or ADG. These correlations mostly align with prior work in yearling aged range sheep [6]. However, Redden et al. used initial body weight whereas we used mid-trial body weights likely leading to the present study detecting correlations between bodyweights and a number of traits, where they detected none. We did not observe a significant correlation between DMI and PWWT EBV in period 2 that was seen in period 1. This could be due to the PWWT EBV representing weight at approximately 120 days of age, whereas the ewes were approximately 355 days of age at the start of period 2.

Between periods 1 and 2, correlations were detected between MTBW (0.691), MBW (0.703), RFI (0.326), and DMI (0.482). These between period correlations can be found in Table 4. It was expected the bodyweights would be correlated between periods and concurs with prior literature [6]. The correlation of RFI and DMI between experimental periods is likely due to similar nutrient composition of the diets used, despite one being pellet form and the other in unprocessed hay. Individual RFI repeatability between periods 1 and 2 was calculated to be 0.157 ± 0.109 indicating RFI is a lowly repeatable trait in range sheep and concurs with published work investigating cattle RFI [27,28]. This may be a result of different physiological state (maintenance versus growth) or slightly different diets fed in each period. While the diets were chemically similar, the physical properties of an alfalfa pellet versus unprocessed forage, could be contributing to an RFI difference between experimental periods. Feeding processed feeds to younger animals is more common in the industry than feeding pellets to older animals closer to maintenance which our study sought to replicate with the different physical forms fed for each period. This low repeatability reinforces that RFI at different physiological stages should be considered different traits in genetic and genomic evaluations even if diets are chemically similar yet physically different. These results also suggest more research is needed to understand the biological mechanisms regulating RFI between growth and maintenance phases in sheep.

### 3.2. Genome-Wide Association

**Figure 1:**
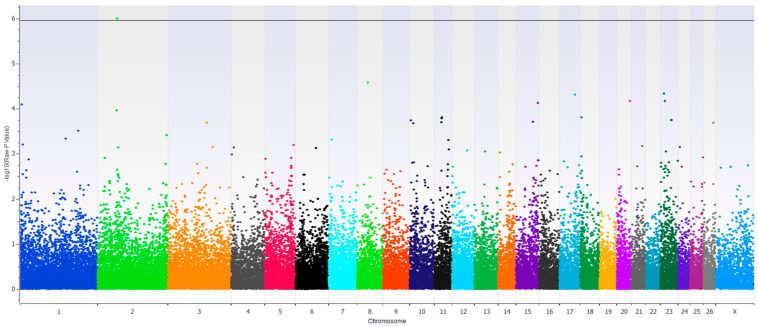
Manhattan plot of −log10 (*p*-values) of the association with experimental period 2 RFI with MTBW as a covariate. The horizontal black line indicates the Bonferroni corrected threshold of 0.05.

A single SNP association was found with the second experimental period RFI and DMI at Oar2:68812505 when MTBW was a covariate in the model (Bonferroni adjusted *p* < 0.05, Figure 1). The associated SNP is located within the gene encoding the Doublesex and Mab-3 Related Transcription Factor 2 (DMRT2). The DMRT family of genes is well known for their primary role in sex determination, however additional roles are continually being identified [29,30,31]. Specifically, Dmrt2 enables DM-domain specific DNA binding activity and can regulate gene expression of other genes such as Myf5 [32,33]. As a part of the Pax3-Dmrt2-Myf5 regulatory cascade which regulates myogenesis in the early stages of the embryo, Dmrt2 plays a role in skeletal muscle development [33]. It also has a role in endochondral bone development as observed in Dmrt2 mouse knockouts which died soon after birth but showed a dwarf phenotype and disrupted rib cage development [34].

In sheep, there have been no direct associations with DMRT2, but MYF5 has been associated with skeletal muscle yield [35]. It is possible that alterations to DMRT2 affect this regulatory cascade and eventual effects of MYF5 on muscle composition of the sheep’s body. This regulatory impact could change the efficiency of the animal if the low RFI animals are maintaining more muscle at the same weight as opposed to fat, since muscle requires less energy per gram of tissue than fat to maintain. However, given the sample population was comprised of ewes not destined to slaughter, we could not test this hypothesis in the current study design. Though there are reports of high feed efficient lambs having less fat content at slaughter providing some evidence for this hypothesis [36].

Cockrum et al. (2012), performed a GWAS for RFI in sheep and found that ovine chromosomes 1, 2, 3, and 18 had the most SNPs associated with RFI and identified *GLIS1*, *IL1RAPL1*, *SOX5*, and *SOX6* as their top candidate genes [19]. Pasandideh et al. (2018), identified *MAGI1*, *ARRDC3*, *PPP4R2*, and *FOXP1* were associated with lower feed efficiency through a GWAS in Baluchi sheep [20]. We did not identify these same genes or similar quantitative trait loci as these previous studies which could be a result from using different breeds, using only ewes, and measuring RFI at different times in the production cycle.

Importantly, small sample size has limited the number of genetic associations that were identified in this study. However, using a conservative Bonferroni multiple testing correction reduces the likelihood the result if spurious. Additional GWAs are needed to validate the current association and bring more resolution to detect additional markers related to RFI during growth and maintenance across sheep breeds and populations. These may also include approaches such as sanger sequencing of DMRT2 to identify non-synonymous mutations, RNA-sequencing to identify possible expression differences or other next generation sequencing technologies to further elucidate the role of DMRT2 in feed efficiency.

## 4. Conclusions

Relying solely on PWWT EBVs to increase growth rates in sheep to reach market weight faster may not be a profitable strategy in rangeland settings. As producers select for higher PWWT EBVs they are increasing the size of their sheep at weaning, but by indirect selection, they are also increasing their flock’s mature body size. Larger ewes have higher DMI which may cause feed costs of the ewe flock to increase as mature size increases. While feed efficiency is not correlated to PWWT EBVs, increased feed requirements of mature ewes may decrease the overall flock profitability depending on the operation. Depending on the lamb price received, producers may need to do a cost-benefit analysis to determine if the larger size is economically sustainable for their operation. While we found a correlation of RFI between growth and maintenance phases, likely attributable to similar diets fed, it was determined to be lowly repeatable and as such should be considered a different trait for optimal genetic evaluation. Further research should investigate other ways to evaluate lambs early in life for predicting future feed efficiency when fed dissimilar diets before the adoption of genomic selection tools using RFI as a phenotype to ensure selection can improve feed efficiency without adverse effects on profitability. The association of *DMRT2* with RFI and DMI indicates that processes early in utero, such as myotome myogenesis and chondrocyte hypertrophy, may play a significant role in feed efficiency and feed intake later in life. However, further work is needed to prove any causal relationship between DMRT2 and RFI and DMI.

## Figures and Tables

**Table 2:** Production Trait Measurements (mean ± SD).

Trait	Experimental Period 1	Experimental Period 2	*p* Value ^1^
Age, days	121 ± 5	355 ± 5	
MTBW kg	27.8 ± 4.39	42.9 ± 7.10	<0.05
MBW kg	14.5 ± 1.70	16.7 ± 2.12	<0.05
ADG kg/day	0.26 ± 0.12	−0.02 ± 0.16	<0.05
DMI kg/day	2.01 ± 0.38	2.02 ± 1.19	0.96
RFI kg/day	0.00 ± 0.24	0.00 ± 0.97	0.92

^1^ *p*-values indicate significant differences between experimental period 1 and experimental period 2 traits using a two tailed *t*-test.

**Table 3:** Matrix of Correlations Between Traits Within Experimental Period One or Two.

	MTBW	MBW	ADG	DMI	RFI	PWWT EBV
MTBW		0.999 *	0.122	0.783 *	−0.003	0.438 *
MBW	0.999 *		0.119	0.785 *	0.000	0.437 *
ADG	0.387 *	0.390 *		0.102	0.000	−0.162
DMI	0.562 *	0.558 *	0.129		0.619 *	0.280 *
RFI	−0.003	−0.009	−0.013	0.820 *		−0.099
PWWT EBV	0.330 *	0.327 *	−0.014	0.210	0.016	

* Correlation tests between traits within experimental period one are above the diagonal and tests between traits within experimental period two are below the diagonal. Statistically significant correlations, *p* < 0.05 are denoted with *.

**Table 4:** Correlation Between Traits in Experimental Period 1 and 2.

Trait	Correlation
MTBW	0.691 *
MBW	0.703 *
ADG	−0.007
DMI	0.482 *
RFI	0.326 *

* *p* values < 0.05 indicate a significant correlation between the same trait in experimental periods one and two.

## Data Availability

The raw data supporting the conclusions of this article will be made available by the authors on request.

## Abbreviations

The following abbreviations are used in this manuscript:

GWAS	Genome-wide association study
MTBW	Mid-test body weight
MBW	Mid-test metabolic body weight
PWWT	Post-Weaning Weight
RFI	Residual Feed Intake
